# RETRACTED: Zhang et al. Hsa-Let-7g miRNA Targets Caspase-3 and Inhibits the Apoptosis Induced by ox-LDL in Endothelial Cells. *Int. J. Mol. Sci.* 2013, *14*, 22708–22720

**DOI:** 10.3390/ijms27114654

**Published:** 2026-05-22

**Authors:** Yefei Zhang, Naiyun Chen, Jihao Zhang, Yaling Tong

**Affiliations:** Emergency Department, First Affiliated Hospital, School of Medicine, Zhejiang University, Hangzhou 310003, China; ychzhd@163.com (N.C.); jhzhangzhd@163.com (J.Z.); yltongzhd@163.com (Y.T.)

The journal retracts the article titled, ”Hsa-Let-7g miRNA Targets Caspase-3 and Inhibits the Apoptosis Induced by ox-LDL in Endothelial Cells” [1], cited above.

Following publication, concerns were brought to the attention of the Editorial Office regarding the integrity of the images, origin of the study and authorship in this article [1].

In accordance with the journal’s standard procedures, the Editorial Office and Editorial Board conducted an investigation that identified indications of inappropriate image editing and duplication between Figure 5E published in ref. [1] and Figure 4E in ref. [2] and presented different experimental conditions. These two papers were under submission at the same time by different authorship groups, which raised further concerns regarding the authenticity, provenance of the data, and authorship. Consequently, the Editorial Board has lost confidence in the reliability of the reported findings and has decided to retract this publication.

This retraction was approved by the Editor-in-Chief of the journal *IJMS*.

The authors have been informed of this retraction but did not provide a comment on this decision.

## References

[1] Zhang Y., Chen N., Zhang J., Tong Y. (2013). RETRACTED: Hsa-Let-7g miRNA Targets Caspase-3 and Inhibits the Apoptosis Induced by ox-LDL in Endothelial Cells. Int. J. Mol. Sci..

[2] Li X., Tian F., Wang F. (2013). Rheumatoid Arthritis-Associated MicroRNA-155 Targets SOCS1 and Upregulates TNF-α and IL-1β in PBMCs. Int. J. Mol. Sci..

